# Hypercalcemia and acute kidney injury caused by occupational inhalation of vitamin D3: a case report

**DOI:** 10.3389/fmed.2026.1932292

**Published:** 2026-09-30

**Authors:** Shan Zhu, Luping Chen, Jun Ying, Hongying Ye, Jian Huang

**Affiliations:** Department of Nephrology, Jinhua Municipal Central Hospital, Jinhua, China

**Keywords:** acute kidney injury, hypercalcemia, inhalation exposure, occupational exposure, vitamin D toxicity

## Abstract

Vitamin D intoxication is a rare but potentially life-threatening condition that can lead to severe hypercalcemia and serious complications. Most reported cases are associated with long-term or high-dose oral vitamin D preparations, dietary supplement use, or inappropriate iatrogenic prescribing. In contrast, occupational inhalation exposure remains exceedingly uncommon. We report the case of a 25-year-old man who developed parathyroid hormone (PTH)-independent hypercalcemia and acute kidney injury (AKI) due to excessive exogenous vitamin D3 exposure in the workplace. Following prompt treatment, his clinical condition improved and he was discharged. This case underscores the importance of actively eliciting a history of occupational exposure in patients with PTH-independent hypercalcemia, particularly when there is no clear history of oral supplement use, to reduce missed diagnoses and facilitate prompt recognition and treatment.

## Introduction

Vitamin D is an essential fat-soluble vitamin and a secosteroid hormone precursor whose metabolites regulate bone metabolism, maintain calcium homeostasis, and modulate diverse biological functions (1, 2). In humans, vitamin D is obtained primarily through two pathways: endogenous cutaneous synthesis after sunlight exposure, accounting for approximately 80% of total vitamin D, and dietary intake from foods such as milk and eggs or from supplements, accounting for approximately 20% (1). Although vitamin D deficiency and insufficiency have emerged as global public health concerns, vitamin D intoxication remains relatively uncommon (3, 4). It is typically associated with prolonged excessive use of over-the-counter supplements or inappropriate iatrogenic prescribing (5, 6). Because routine history-taking often focuses on medication use and dietary supplement intake, diagnosis may be delayed when patients do not spontaneously report relevant occupational exposure.

We report the case of a previously healthy young man who developed severe hypercalcemia and acute kidney injury following substantial occupational inhalation exposure to vitamin D3 powder. The pivotal diagnostic turning point was the decision to move beyond the conventional paradigm of supplement overdose and systematically re-evaluate the patient's occupational history, which ultimately identified inhalational vitamin D3 intoxication as a rare etiology. This report highlights the importance of incorporating a detailed occupational exposure history into the routine etiological assessment of patients with unexplained parathyroid hormone-independent hypercalcemia, thereby reducing missed diagnoses, preventing diagnostic delays, and facilitating timely intervention.

## Case presentation

A 25-year-old man presented to a local hospital with a one-week history of anorexia and fatigue, followed by nausea and vomiting for two days. Laboratory tests revealed elevated serum creatinine (Scr) at 167.1 *μ*mol/L (reference range, 40–128 *μ*mol/L), blood urea nitrogen (BUN) at 9.81 mmol/L (reference range, 2.8–8.0 mmol/L), and urinary white blood cells at 42/*μ*L (reference range, 0–8/μL), while routine hematologic and liver function tests were unremarkable. He was transferred to our hospital later that day for further evaluation and management.

His medical, personal, and family histories were unremarkable. Upon admission, his vital signs were stable. Physical examination revealed no abnormalities of the cardiovascular, respiratory, or abdominal systems, and no peripheral edema was observed. Initial laboratory tests demonstrated elevated Scr levels of 189.0 μmol/L (reference range, 40.0–135.0 μmol/L) and marked hypercalcemia, with a serum calcium concentration of 3.52 mmol/L accompanied by suppressed PTH levels of 7.4 pg/mL. Urinalysis revealed an increased urinary white blood cell count of 55.2/μL (reference range, 0–25.0/μL). Twenty-four-hour urinary protein excretion was mildly elevated at 0.205 g (reference range, <0.15 g/24 h), and the urinary albumin-to-creatinine ratio was increased to 43.41 mg/g (reference range, 0–22.10 mg/g). Urine sediment examination showed no significant abnormalities in urinary red blood cells, casts, or crystals, and urine culture was negative. The complete laboratory findings are summarized in Table 1.

**Table 1:** Initial laboratory investigations.

Laboratory investigations	Patient value	Reference value
Serum creatinine (μmol/L)	189.0	40.0–135.0
Blood urea nitrogen (mmol/L)	11.40	3.20–7.14
Sodium (mmol/L)	148.8	137.0–147.0
Potassium (mmol/L)	4.06	3.50–5.30
Calcium (mmol/L)	3.52	2.08–2.60
Albumin (g/L)	47.8	40.0–55.0
PTH (pg/mL)	7.4	12.0–88.0
25(OH)D (ng/mL)	>150	20–100
24-hour urinary calcium (mmol/24 h)	10.95	2.50–7.50
24-hour urinary protein (g/24 h)	0.205	< 0.15
Urinary albumin-to-creatinine ratio (mg/g)	43.41	0–22.10
Urine white blood cells (/μL)	55.2	0–25.0
Urine red blood cells (/μL)	15.7	0–23.0
Urinary casts (/μL)	0.84	0–1.00
Urinary crystals	negative	negative
Urine culture	negative	negative

PTH, parathyroid hormone; 25(OH)D, 25-hydroxy vitamin D.

To further clarify the etiology of hypercalcemia, additional diagnostic investigations were undertaken. Given the presence of severe PTH-independent hypercalcemia, a comprehensive evaluation was performed to exclude secondary causes, including malignancy and granulomatous diseases. Immunofixation electrophoresis, serum immunoglobulins, serum free light chains, and tumor markers showed no significant abnormalities. Chest and abdominal computed tomography, bone scintigraphy, and parathyroid ultrasonography were also unremarkable. Notably, serum 25-hydroxyvitamin D [25(OH)D] was markedly elevated at >150 ng/mL.

Subsequently, we systematically asked about his medical history again and confirmed that he had not recently used any prescription or over-the-counter medications, vitamin D preparations, calcium supplements, or other nutritional supplements. Further investigation of his occupational history revealed that he had worked as a workshop operator in a vitamin D3 production facility for approximately 2–3 months before symptom onset. His duties included transferring semi-finished vitamin D3 products into acetone-containing reaction vessels for purification. After purification and automated drying, he was responsible for packaging the dried vitamin D3 powder into bags. These procedures were performed every 2–3 days, with each exposure episode lasting approximately 30 min. The factory provided basic personal protective equipment, including safety goggles, rubber gloves, and dust masks. However, detailed information regarding the specifications, fit, and actual use of the respiratory protective equipment was unavailable. In addition, no environmental dust monitoring or personal exposure assessment data were available, and reliable information on engineering controls, such as local exhaust ventilation or enclosed powder-handling systems, could not be obtained. Therefore, the airborne concentration of vitamin D3 powder and the effectiveness of the occupational protective measures could not be quantitatively evaluated. Despite mask use during these operations, repeated occupational exposure to vitamin D3 powder was considered highly consistent with the markedly elevated serum 25(OH)D level, suppressed PTH, hypercalcemia, and AKI observed in this patient.

After the diagnosis was established, the patient received intravenous hydration with 0.9% sodium chloride at a total daily volume of approximately 2,000–2,500 mL. Because serum calcium did not decline significantly with initial fluid therapy, intravenous furosemide (20 mg once daily) and subcutaneous salmon calcitonin (100 IU every 12 h) were subsequently administered for four consecutive days. Intravenous pantoprazole (40 mg daily) was given for gastric mucosal protection. Additional supportive measures included a low-calcium diet, close monitoring of vital signs, encouragement of adequate oral hydration, and symptomatic treatment of nausea. Serial biochemical monitoring during hospitalization showed progressive decreases in serum calcium and creatinine levels, both of which eventually returned to the normal range. Trends in Scr and calcium over time are summarized in Figure 1.

**Figure 1:**
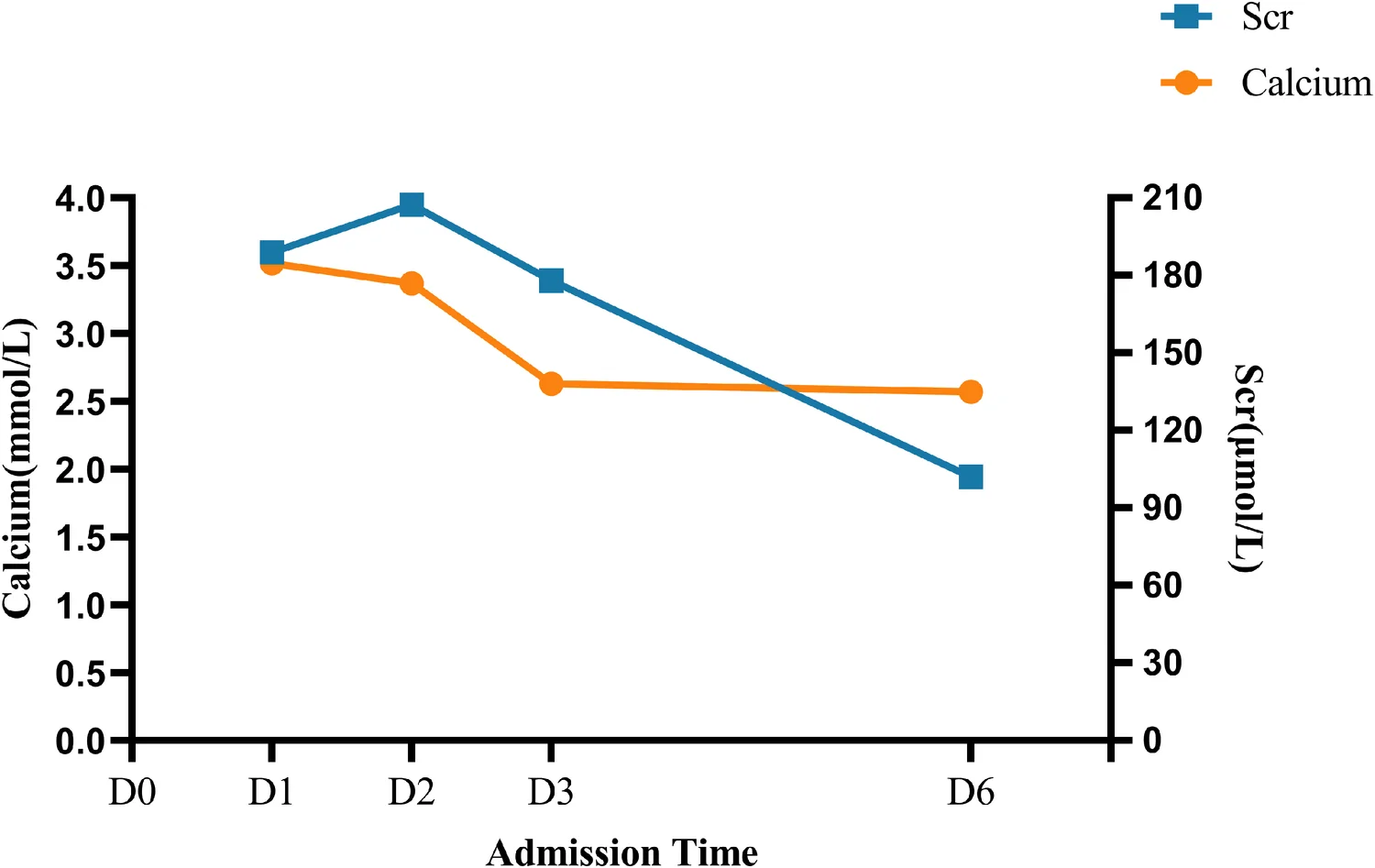
Trends in Scr and calcium during hospitalization.

## Discussion

Hypercalcemia is a clinically significant electrolyte disturbance that requires timely identification and management. Based on total serum calcium concentrations, it is commonly categorized as mild (<3.0 mmol/L), moderate (3.0–3.5 mmol/L), or severe (≥3.5 mmol/L) (7). Its etiologies are diverse and are broadly classified as PTH-dependent or PTH-independent. Primary hyperparathyroidism is the predominant cause of PTH-dependent hypercalcemia, whereas malignancy, vitamin D-mediated disorders, granulomatous diseases, medications, and several less common endocrine or metabolic conditions should be considered when PTH is appropriately suppressed (8). In the present case, the serum calcium concentration was 3.52 mmol/L, accompanied by a markedly suppressed PTH level of 7.4 pg/mL and elevated serum 25(OH)D concentration (>150 ng/mL), directing the diagnostic evaluation toward PTH-independent hypercalcemia due to vitamin D intoxication. The patient predominantly presented with anorexia, nausea, vomiting, and fatigue, consistent with gastrointestinal involvement and systemic manifestations of hypercalcemia. Nekoukar et al. reported vitamin D3 intoxication following accidental oral overdose in a young man who presented with gastrointestinal and systemic symptoms similar to those observed in our patient (9). However, unlike our patient, he did not develop hypercalcemia or renal impairment. This comparison highlights the heterogeneous clinical spectrum of vitamin D intoxication and underscores the importance of considering diverse exposure routes and individual susceptibility during clinical assessment. The parallel improvement in serum calcium and renal function after treatment further suggests that hypercalcemia played an important role in the development of the acute kidney injury.

The most unusual and clinically important feature of this case was the identification of occupational exposure as the probable source of vitamin D3 excess. Most previously reported cases of vitamin D intoxication have been associated with long-term or high-dose use of vitamin D preparations, over-the-counter dietary supplements, prescription dosing errors, or misuse of fortified foods (6, 10–15). The patient denied the use of medications or nutritional supplements. Further inquiry into his occupational history revealed that he worked in the production workshop of a vitamin D3 manufacturing facility and had repeated direct contact with vitamin D3 powder before symptom onset. Therefore, occupational inhalational exposure was considered the most likely source of vitamin D3 intoxication. If history-taking had been limited to medications and nutritional supplements, this etiological clue could easily have been overlooked. The mechanism of hypercalcemia induced by inhaled vitamin D3 is shown in Figure 2.

**Figure 2:**
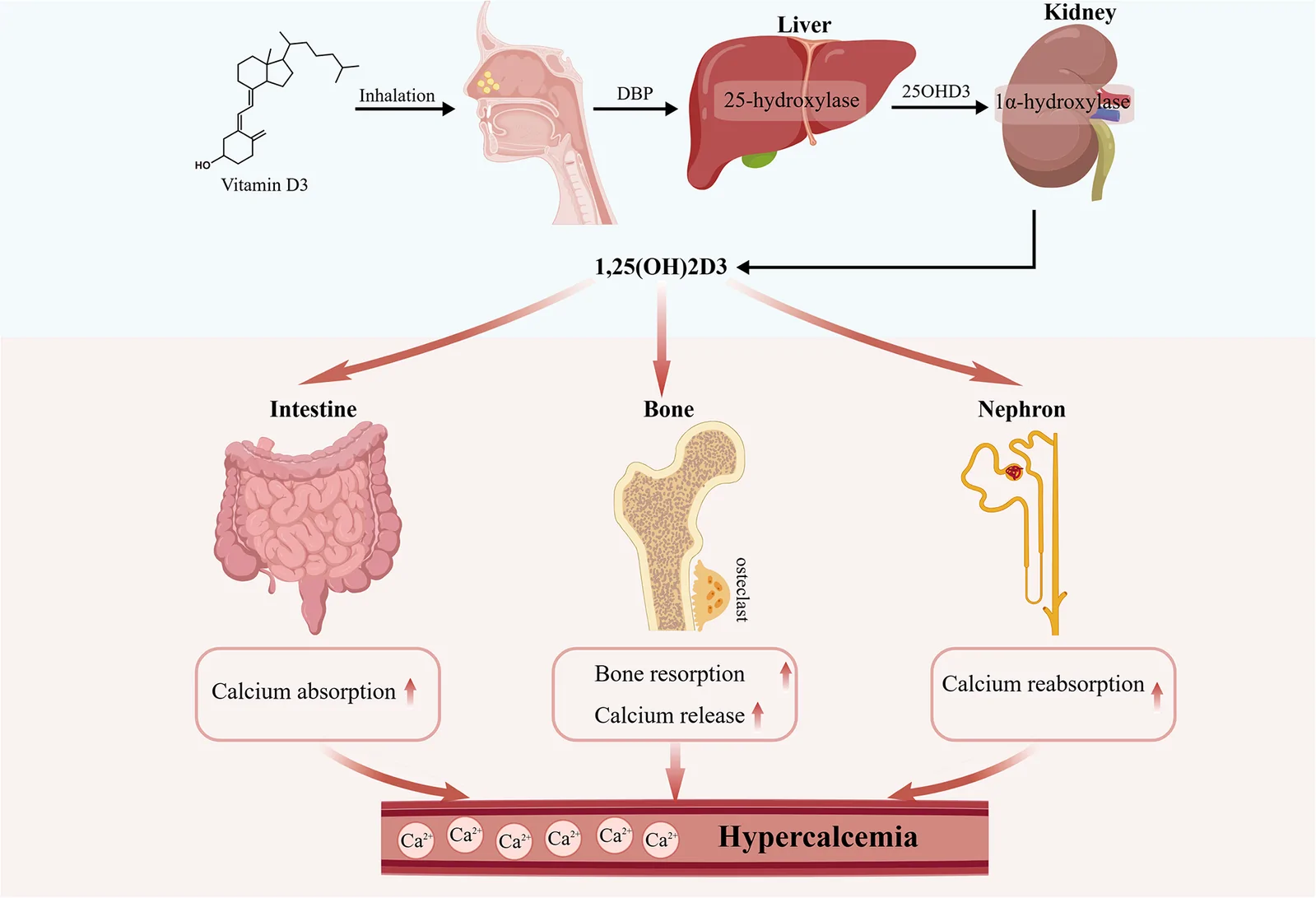
The mechanism of hypercalcemia induced by inhaled vitamin D3 (created with BioGDP.com) (29); DBP, vitamin D binding protein; 25(OH)D3, 25-hydroxyvitamin D; 1,25(OH)2D3, 1,25-dihydroxyvitamin D3; Ca^2+^, calcium. Created with www.BioGDP.com.

Inhalation is one of the most important routes of exposure in occupational environments (16). Airborne powder particles may deposit throughout the respiratory tract depending on their aerodynamic diameter, breathing pattern, and workplace conditions. The exceptionally thin alveolar–capillary membrane, combined with its vast surface area for diffusion, enables the lungs to rapidly absorb exogenous chemicals with diverse physicochemical properties into the systemic circulation. This may represent one of the key mechanisms underlying the pulmonary absorption of vitamin D3 (17). As a lipophilic molecule, vitamin D3 may theoretically be absorbed through the nasal and respiratory mucosa or through the gastrointestinal tract after dust particles deposited in the upper airway are swallowed (18). The nasal and respiratory mucosa are richly vascularized and possess relatively thin epithelial barriers, facilitating the passive diffusion of certain lipophilic substances into the systemic circulation (19). In recent years, intranasal and inhalational delivery have gained increasing attention as noninvasive routes of drug administration because they may bypass hepatic first-pass metabolism, enhance local delivery, and improve the bioavailability of selected agents (20, 21). Studies have also demonstrated that vitamin D3 metabolites can be delivered to the respiratory tract by inhalation, producing both local and systemic effects (22–24). These mechanisms could potentially contribute to markedly elevated serum 25(OH)D levels and subsequently precipitate severe hypercalcemia and AKI.

This case also has important implications for occupational prevention. At the process level, powder-handling operations should be enclosed or automated whenever technically feasible. Local exhaust ventilation and effective dust-collection systems should be installed at powder-transfer, drying, and packaging points to minimize airborne particulate generation. Workplace air monitoring and, where feasible, personal exposure assessment should be conducted to identify high-exposure tasks and evaluate the effectiveness of engineering controls (25). Respiratory protection should be regarded as a complementary measure and should include appropriate selection of particulate respirators, fit testing for tight-fitting devices, proper donning and use, routine maintenance, and regular worker training in accordance with applicable occupational respiratory-protection standards (26).

This case has several limitations. First, the limitation of this case is absence of serum 25(OH)D measurement at discharge and post-discharge biochemical follow-up. Because vitamin D is a lipophilic compound that is stored in adipose tissue and has a slow turnover rate, accumulated vitamin D may persist after cessation of exposure and potentially contribute to prolonged or recurrent hypercalcemia (27, 28). Consequently, the long-term course, including whether serum calcium remained stable or whether rebound hypercalcemia occurred, could not be determined. In addition, serum 1,25-dihydroxyvitamin D was not measured, and the serum 25(OH)D level was reported as >150 ng/mL because it exceeded the upper limit of the assay. These factors preclude a more precise assessment of the severity of vitamin D intoxication. Only two workers were involved in the relevant production process, and clinical or biochemical screening data for the other worker were unavailable. Therefore, it remains unclear whether the patient was truly the only affected individual, and asymptomatic or subclinical exposure in the other worker cannot be excluded. Furthermore, workplace air sampling and personal exposure monitoring were unavailable. Thus, the airborne concentration of vitamin D3, cumulative exposure dose, and potential dose–response relationship could not be quantified.

In summary, in patients with hypercalcemia and suppressed PTH, vitamin D intoxication should not be prematurely excluded simply because routine history-taking reveals no intake of vitamin D or calcium supplements. Clinicians should further inquire about occupational history, working conditions, dust exposure, protective measures, and post-exposure management. More broadly, this case highlights the need for improved engineering controls, appropriate respiratory protection, environmental exposure monitoring, and occupational health surveillance in workplaces handling highly concentrated vitamin D3 powders.

## Conclusion

Occupational inhalation of vitamin D3 powder may represent a rare cause of exogenous vitamin D intoxication, leading to PTH-independent hypercalcemia and acute kidney injury. This case highlights the importance of considering occupational exposures in the differential diagnosis of hypercalcemia and acute kidney injury. Employers should strengthen occupational health management, refine operating procedures for enclosed-space work, implement effective dust-control measures, and provide training in personal respiratory protection to reduce the risk of similar occupational intoxication events.

## Data Availability

The original contributions presented in the study are included in the article/Supplementary Material, further inquiries can be directed to the corresponding author.
